# Supplementary material for: The Evolution and Characterization of the RNA Interference Pathways in Lophotrochozoa
Source: Genome Biol Evol. 2024 May 7;16(5):evae098. doi: 10.1093/gbe/evae098 (PMC11114477; doi:10.1093/gbe/evae098)
Supplement: evae098_Supplementary_Data [file evae098_supplementary_data.zip › Supplementary_materials.zip!/selected_miRNAs/DaRe_selected_miRNas/DaRe_NC_007120.7_22295.pdf]

Provisional ID	:	NC_007120.7_22295
Score total	:	46.3
Score for star read(s)	:	3.9
Score for read counts	:	39.4
Score for mfe	:	2
Score for randfold	:	1.6
Score for cons. seed	:	-0.6
Total read count	:	89
Mature read count	:	65
Loop read count	:	0
Star read count	:	24

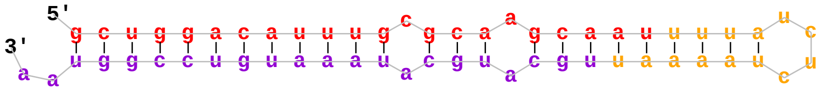

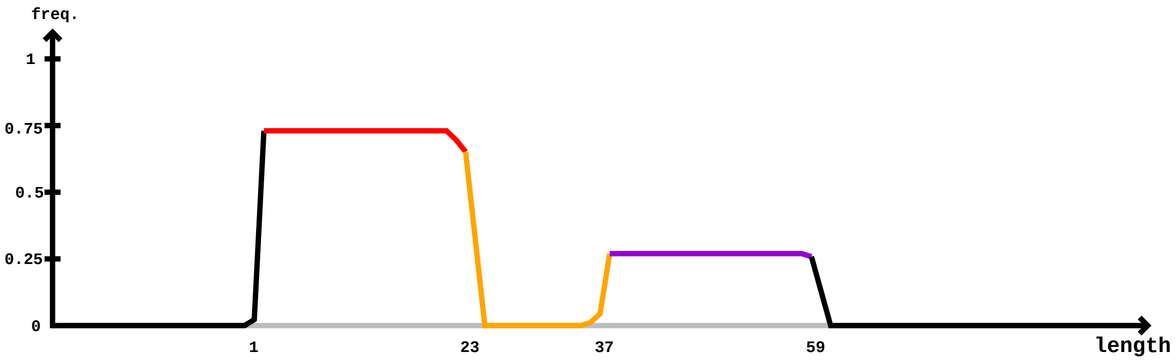

Mature Star

```
5'- ugguuuuugguuugauguucgcuggacauuugcgcaagcaauuuuaucucuaaaauugcaugcauaaauguccgguaaacaucaaauuaaaucugcaucuuacaucaucugc   -3'   obs
    ugguuuuugguuugauguucgcuggacauuugcgcaagcaauuuuaucucuaaaauugcaugcauaaauguccgguaaacaucaaauuaaaucugcaucuuacaucaucugc         exp
    ((...((((((((((((((.((((((((((((.(((.(((((((((....))))))))).))).)))))))))))).))))))))))))))....))...............    reads   mm   sample
    ...................cgcuggacauuugcgcaagcaau......................................................................    1       0    Ov3
    ....................gcuggacauuugcgcaGgcaa........................................................................    1       1    Ov3
    ....................gcuggacauuugcgcaagcaa........................................................................    1       0    Ov3
    ....................gcuggacauuugcgcaagcaau......................................................................    19      0    Ov3
    ....................gcuggacauuugcgcaGgcaau......................................................................    9       1    Ov3
    ....................gcuggacauuugcgcaagcaauu.....................................................................    2       0    Ov3
    .......................................................uugcaugcauaaauguccgguaa..................................    1       0    Ov3
    ........................................................ugcaugcauaaauguccggua...................................    1       0    Ov3
    ........................................................ugcaugcauaaauguccgguaC..................................    4       1    Ov3
    ........................................................ugcaugcauaaauguccgguaCa.................................    1       1    Ov3
    ........................................................ugcaugcauaaauguccgguaaa.................................    1       0    Ov3

    ....................gcuggacauuugcgcaagcaau......................................................................    1       0    Gu1

    ...................cgcuggacauuuAcgcaagcaau......................................................................    1       1    Gu2
    ....................gcuggacauuugcgcaagcaa........................................................................    1       0    Gu2
    ....................gcuggacauuugcgcaagcaau......................................................................    2       0    Gu2

    ....................gcuggacauuugcgcaagcU........................................................................    3       1    Ov2
    ....................gcuggacauuugcgcaGgcaau......................................................................    7       1    Ov2
    ....................gcuggacauuugcgcaagcaau......................................................................    5       0    Ov2
    ......................................................auugcaugcauaaauguccgguaC..................................    1       1    Ov2
    .......................................................uugcaugcauaaauguccgguaC..................................    2       1    Ov2
    ........................................................ugcaugcauaaauguccgguaU..................................    1       1    Ov2
    ........................................................ugcaugcauaaauguccgguaa..................................    1       0    Ov2
    ........................................................ugcaugcauaaauguccgguaC..................................    6       1    Ov2

    ....................gcuggacauuugcgcaagcaa........................................................................    1       0    Ov1
    ....................gcuggacauuugcgcaagcaau......................................................................    8       0    Ov1
    ....................gcuggacauuugcgcaagcaaA......................................................................    1       1    Ov1
    ....................Ccuggacauuugcgcaagcaau......................................................................    1       1    Ov1
    ....................gcuggacauuugcgcaGgcaauu.....................................................................    1       1    Ov1
    ........................................................ugcaugcauaaauguccgguaU..................................    1       1    Ov1
```

```
                    Mature                                Star
ugguuuuugguuugauguucgcuggacauuugcgcaagcaauuuuaucucuaaaauugcaugcauaaauguccgguaaacaucaaauuaaaucugcaucuuacaucaucugc
........................................................ugcaugcauaaauguccgguaa..................................    1    0    Ov1
........................................................ugcaugcauaaauguccgguaC..................................    3    1    Ov1
```
